# Rose Rosette Disease Resistance Loci Detected in Two Interconnected Tetraploid Garden Rose Populations

**DOI:** 10.3389/fpls.2022.916231

**Published:** 2022-07-07

**Authors:** Jeekin Lau, Ellen L. Young, Sara Collins, Mark T. Windham, Patricia E. Klein, David H. Byrne, Oscar Riera-Lizarazu

**Affiliations:** ^1^Department of Horticultural Sciences, Texas A&M University, College Station, TX, United States; ^2^Department of Entomology and Plant Pathology, University of Tennessee Institute of Agriculture, Knoxville, TN, United States

**Keywords:** *Rose rosette emaravirus*, rose rosette virus, emaravirus, quantitative trait loci, *Rosa*, eriophyid mite, *Phyllocoptes fructiphilus*

## Abstract

Rose rosette disease (RRD), caused by the *Rose rosette emaravirus* (RRV), is a major threat to the garden rose industry in the United States. There has been limited work on the genetics of host plant resistance to RRV. Two interconnected tetraploid garden rose F_1_ biparental mapping populations were created to develop high-quality tetraploid rose linkage maps that allowed the discovery of RRD resistance quantitative trait loci (QTLs) on linkage groups (LGs) 5, 6, and 7. These QTLs individually accounted for around 18–40% of the phenotypic variance. The locus with the greatest effect on partial resistance was found in LG 5. Most individuals with the LG 5 QTL were in the simplex configuration; however, two individuals were duplex (likely due to double reduction). Identification of resistant individuals and regions of interest can help the development of diagnostic markers for marker-assisted selection in a breeding program.

## Introduction

The modern-day garden rose is the result of interspecific crosses between roughly 10 different species resulting in a crop, which is highly heterozygous in nature (Gudin, 2000). Commercial garden roses are a complex of diploid (2*n* = 2*x* = 14), triploid, and tetraploid hybrids (Zlesak, 2007). One *Rosa* species has been reported to be a decaploid (Jian et al., 2010). Roses are vegetatively propagated *via* their own root cuttings or budded onto rootstock cultivars. Roses are generally outcrossing with varying levels of self-incompatibility but can be self-pollinated and sib mated. Depending on the level of inbreeding, roses also manifest inbreeding depression. The multitude of species used in its development, the varying ploidy levels, and high heterozygosity make variety development in roses particularly challenging.

Rose rosette disease (RRD), caused by the *Rose rosette emaravirus* (RRV) (Laney et al., 2011), is threatening the United States garden rose (*Rosa hybrida* L.) industry which had $168 million in sales in 2019 (USDA NASS, 2019). This virus is a negative-sense RNA emaravirus (Mielke-Ehret and Mühlbach, 2012), which is vectored by a microscopic eriophyid mite (*Phyllocoptes fructiphilus* Keifer) (Allington et al., 1968; Amrine et al., 1988). The virus is transmitted *via* the mite and grafts but not by mechanical means (pruners) (Doudrick et al., 1987; Amrine et al., 1988).

Although symptoms of RRD were first described in the early 1940s, on *Rosa woodsii* Lindl. in the United States (Conners, 1941; Amrine, 1996), only in the past decade has the virus become a problem in commercial, public, residential rose plantings, and in commercial production facilities. Symptoms include large masses of reddish prolific twisted growth extending from an otherwise healthy-looking bush. These prolific growths (rosettes) are identified by their strapped (long and thin) leaves accompanied by shorter internodes and increased thorniness on cultivars with prickles (Windham et al., 2014). The early symptoms of RRD before the witches’ broom include (1) strapped leaves, (2) flattened stems, and (3) increased thorniness. If two of these symptoms are present, the plant is likely infected (Windham et al., 2019).

The excessive new growth of the plant expends the plant’s energy resources causing death in 3–4 years. Infected plants showing rosetting are not visually pleasing and need to be removed as they serve as a source of viral inoculum. Although detection and relative quantification of RRV viral titer can be performed using reverse transcription-quantitative polymerase chain reaction (RT-qPCR) (Dobhal et al., 2016), RRD is a difficult disease to phenotype as it can take up to 3–4 years to properly assess the vulnerability of any given genotype. Other complications include asymptomatic genotypes with a detectable virus but no symptoms. There is no cure for the viral infection which typically results in the death of the plant within 3–4 years (Pemberton et al., 2018). Thus, breeding for resistance is of upmost importance for the wellbeing of the rose industry in North America (Byrne et al., 2018).

To the best of our knowledge, there has been no previous genetic work assessing the genetic factors affecting resistance to RRD caused by RRV in tetraploid roses. Thus, we created two tetraploid garden rose F_1_ populations with the purpose to (1) conduct quantitative trait locus (QTL) scans to identify regions of the genome associated with resistance to RRD and (2) use results from QTL analysis to identify individuals and ultimately haplotypes that carry favorable alleles for resistance to use in future breeding for resistance to RRD.

## Materials and Methods

### Population Development and Phenotyping

Two tetraploid garden rose F_1_ populations, *Rosa* L. “ORAfantanov” (Stormy Weather™) × *Rosa* L. “Radbrite” (Brite Eyes™) (SWxBE) *n* = 200 and *Rosa* L. “Radbrite” (Brite Eyes™) x *Rosa* L. “BAIgirl” (Easy Elegance^®^ My Girl) (BExMG) *n* = 157, were created for studying rose rosette resistance in 2016 as a joint effort between the Texas A&M University Rose Breeding and Genetics Program and Weeks Roses. Due to the number of clonal replicates available, only 175 and 125 genotypes, respectively, were screened for rose rosette resistance while the full number of individuals was used for linkage mapping. In previous cultivar resistance trials in Tennessee, the parents Brite Eyes and Stormy Weather were rated as moderately susceptible while My Girl was extremely susceptible (personal communication).

The mapping populations were propagated *via* rooted cuttings in 2017 and planted in a randomized complete block design with two blocks (with a single plant as the experimental unit) at the University of Tennessee AgResearch Plateau Research and Education Center in Crossville, TN (36.01, -85.13). The plants were planted in double row beds (four feet between rows) at a spacing of four feet between plants and a distance between beds of 10 feet. Drip irrigation and fertilizer were used as needed, and a mulch consisting of wood and bark chips was used for weed control. The soil type at this location is a Lily Loam. Natural RRD infections from infected wild roses (*Rosa multiflora* Thumb.) surrounding the field served as a source of inoculum. Natural infection was augmented by planting inoculum rows with RRD infected plants on the outside rows and in the middle of the field. Further infection augmentation was performed by clipping symptomatic rosettes collected from infected plants onto healthy plants once per growing season (year). These rosettes were checked for the presence of the viral vectoring mite prior to clipping. Plants were scored for disease severity using a 0–3 scale. Severity was rated 0 = no symptoms, 1 = small single shoot with rosetting, 2 = 2–3 shoots with rosetting, and 3 = 4 or more shoots with rosetting (personal communication). The plants were scored once yearly in 2019 and 2020. In 2021, the populations were visually scored twice for RRD, once in September, and later in November. The last set of observations (in November) were taken on a scale of 0–5 where 0 was no severity, 1 = 0–10% of the plant symptomatic, 2 = 11–25%, 3 = 26–50%, 4 = 51–75%, and 5 = > 75%. This was later scaled to match the 0–3 scale used previously. Variance components were estimated from a completely random model using ASReml-R version 4.1 (Butler et al., 2009):


$$y_{ijk}=\mu +G_{i}+E_{j}+R_{k\left(j\right)}+GE_{ij}+\varepsilon_{ijk}$$

where *y*_*ijk*_ is the phenotypic value of genotype *i* at environment (year) *j* in block *k*; μ is the overall mean; *G*_*i*_ is the effect of the genotype *i*; *E*_*j*_ is the effect of the environment *j* (in our case the observation set); *R*_*k(j)*_ is the effect of block nested within the environment; *GE*_*ij*_ is the genotype by environment effect; and ε_*ijk*_ is the residual defined as the interaction between plot and correlation of years (to account for the longitudinal characteristic of this dataset). For estimating best linear unbiased estimates (BLUEs) used for QTL scans, the effect of genotype was considered fixed while the rest of the model was considered random. From the variances estimated in the mixed model above, we estimated the proportion of genetic, environmental, and genetic by environmental variance attributed to the phenotypic variance. Least-square means (LS means) were calculated for each family by year separately and across years. LS means were compared using a Student’s *t*-test (α = 0.05). Spatial effects were checked by looking at heatmaps and adding row, plant, and row by plant interaction as fixed effects to the model and checking for significance using Wald’s test.

Viral presence and relative quantification were performed using RT-qPCR as reported by Dobhal et al. (2016). Two positive controls (symptomatic plant and synthetic positive) and two negative controls (water and healthy plant sample) were used during RT-qPCR screenings. Due to the number of genotypes screened and the labor involved, once a genotype tested positive for the virus, no subsequent RT-qPCR testing was conducted. Genotypes that were negative for virus presence were tested until either virus was detected or the end of the experiment. The lowest cycle threshold (*C*_*t*_) value for each genotype at the end of the experiment was used for QTL mapping.

### Genotyping, Linkage Mapping, and Quantitative Trait Loci (QTL) Mapping

Unexpanded young leaf tissue was collected, flash-frozen in liquid nitrogen, and stored at −80°C until DNA extraction. DNA was extracted using a CTAB protocol (Yan et al., 2018). Extracted DNA samples were incubated with RNase at 37°C and purified using the OneStep™ PCR Inhibitor Removal Kit (Zymo Research, Irvine, CA, United States). Extracted DNA samples were quantified using a DS-11 spectrophotometer (DeNovix Inc., Wilmington, DL, United States) using Accublue^®^ high dsDNA Quantification standards (Biotium, Inc., Fremont, CA, United States). DNA samples with a concentration of greater than 50 ng/μl were sent to Thermo Fisher Scientific (Waltham, MA, United States) for genotyping on the Axiom WagRhSNP 68k array (Koning-Boucoiran et al., 2015). Raw genotyping light intensity data were summarized using the “Summarized Signal Intensity” workflow inside of Axiom Analysis Suite (version 4.1; Affymetrix, Inc.) and R package fitPolyTools (version 1.1.1; unpublished package by Roeland Voorrips.). Marker allele dosage was called using the default parameters of the “saveMarkerModels” function indicating ploidy as tetraploid and population structure using the “pop.parents” and “population” arguments of the R package fitPoly (version 3.0.0) (Voorrips et al., 2011). Each SNP on the WagRhSNP 68k array has two probes, one for the forward and the other for the reverse strand. Thus, a custom R script was written to combine data from the two probes. Probes with the same genotype call and those with only one probe that was called were kept, whereas probes that had differing genotype calls were discarded. The custom script has since been converted into the “compare_probes” function in an R-package RoseArrayTools hosted at https://github.com/jeekinlau/RoseArrayTools.

For the analysis of each population individually, linkage mapping was performed using the R software package polymapR (version 1.1.2) (Bourke et al., 2017a). Markers were filtered only allowing 1% missing data per marker and 1% missing data per individual. Pairwise recombination fractions were estimated between all the simplex × nulliplex markers and clustering these markers using the recombination fraction matrix created a scaffold of 7 chromosomes and 28 homologs. Recombination fractions were calculated between all other marker types and the markers on the scaffold and fitted to the scaffolds. Marker ordering was performed *via* MDSmap as implemented in polymapR using the Haldane mapping function. After linkage map construction, preferential pairing is detected using closely linked repulsion phase simplex × nulliplex markers using the test_prefpairing function in polymapR. This function allows for the reestimation of linkage of accounting for preferential pairing. The resulting maps were compared with the *Rosa chinensis* rose genome (Hibrand-Saint Oyant et al., 2018) to check for mapping quality. Markers on the Axiom WagRhSNP 68k were BLASTed against the rose genome assembly to obtain the genome position of the markers. Marker statistics were generated using the R shiny app The Genetic Map Comparator (Holtz et al., 2017).

From phased maps, genotype and homolog probabilities were calculated using the R software package MAPpoly (version 0.3.0) (Mollinari and Garcia, 2019). These genotype probabilities were used for QTL scans in the R software package QTLpoly (version 0.2.3) (da Silva Pereira et al., 2020) with a random-effect multiple QTL mapping method (REMIM) using a genome-wide forward significance of 0.20 and a backward elimination significance of 0.05. QTLpoly calls QTLs that are very close to statistical significance as “putative” QTLs. It is important to note that regardless of QTLpoly’s use of the term “putative,” all the QTLs discovered in this study have not been validated beyond the context of this study. Thus, all QTLs discovered in this study are considered putative. To avoid confusion and to be consistent with other QTLpoly users, the term “putative” used in the rest of this study refers to QTLpoly’s QTL significance classification.

In addition to QTL analysis of each biparental mapping population separately, we took another approach, which harnessed the interconnectedness of our two mapping populations. The Julia software package PolyOrigin (version 0.5.10) (Zheng et al., 2021) was used to reconstruct the parental haplotypes of all the interconnected progenies. The joint family QTL analysis was performed using the R software package diaQTL (version 1.04) (Amadeu et al., 2021). The strength of this approach is that more individuals are used in the joint analysis, thus providing better power for the detection of genetic factors inherited from the common parent.

## Results

### Phenotypic Evaluations

**FIGURE 1:**
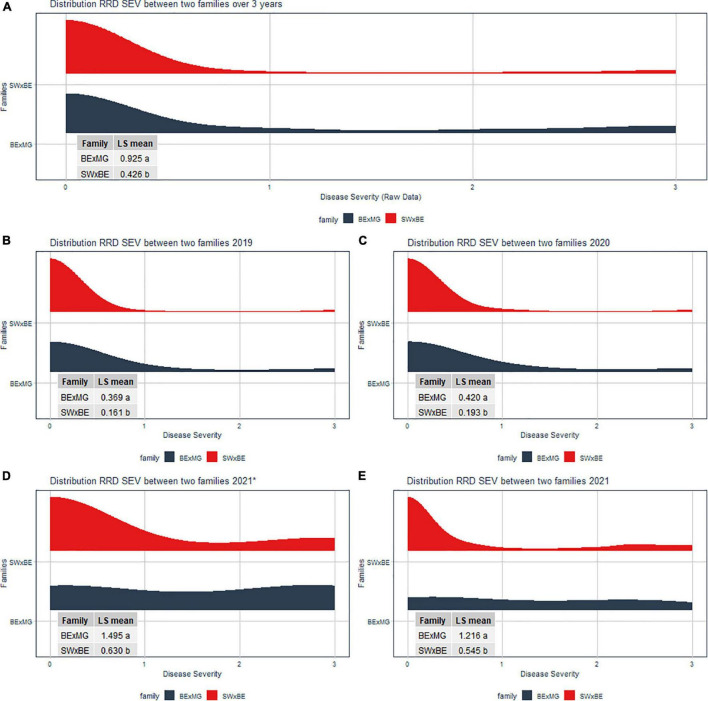
Half violin plots of the raw rose rosette disease (RRD) severity ratings over 3 years separated by the two families. Least-squared means were compared via a *t*-test. The panes of the violin plots are **(A)** RRD severity distribution of over all 3 years (2019 to 2021), **(B)** RRD severity distribution in 2019, **(C)** RRD severity distribution in 2020, **(D)** RRD severity distribution in September 2021, and **(E)** RRD severity distribution in November 2021.

The RRD severity BLUEs and the *C*_*t*_ value from RT-qPCR were moderately negatively correlated (*r* = −0.601, *P* < 0.001) as an increase in RRD symptoms is thought to be correlated with higher viral load, represented by lower PCR cycles needed to detect the viral presence. The ratio of individuals with susceptible ratings to RRD was greater in the BExMG population than in the SWxBE population (Table 1 and Figure 1). Overall and separately by year, the susceptible observations were roughly double in the BExMG population, suggesting that there may be more genetic factors in the SWxBE population that are responsible for RRD resistance when compared with the BExMG population. For the years 2019 and 2020 (Figures 1B,C), there were many severity scores of zero and few susceptible scores. However, in the year 2021 (Figures 1D,E), there were many more observations that were scored as susceptible (1–3) when compared with the earlier years. RRD takes a few years for infection and symptoms to begin to show in new plantings. The difference in disease incidence from year to year can be seen in Figures 1B–E, as there is an increase in susceptible ratings over the years as the virus has had time to infect the plants. Heatmaps show that disease throughout the field was uniform (Supplementary Figure 1) and neither row, plant, nor interaction effects were significant. Thus, there is no spatial variation affecting disease.

**TABLE 1:** Ratios of rose rosette disease susceptible to resistant observations in tetraploid garden roses phenotyped in Crossville, TN over 3 years.

Family	Ratio of visual phenotypic evaluations that were susceptible				
	Overall (2019–2021)	2019	2020	2021a	2021b
SWxBE	0.167	0.077	0.100	0.248	0.291
BExMG	0.391	0.181	0.230	0.586	0.642

*The ratio was calculated by taking the number of observations that were susceptible (ratings = 1, 2, or 3) divided by all observations (ratings = 0, 1, 2, and 3). Two sets of observations were taken in 2021 once earlier in the year 2021a (September), and once later in the year 2021b (November). SWxBE = Stormy Weather × Brite Eyes. BExMG = Brite Eyes × My Girl.*

### Linkage Mapping

**FIGURE 2:**
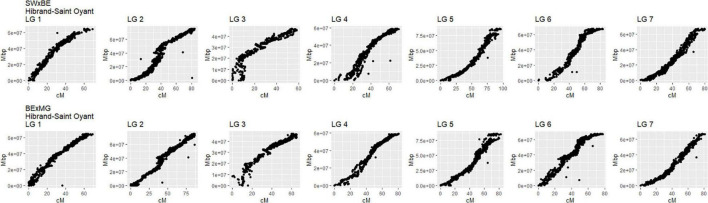
Linkage maps of two tetraploid garden rose mapping populations, Stormy Weather × Brite Eyes (SWxBE) and Brite Eyes by My Girl (BExMG) aligned to the *Rosa chinensis* reference genome (Hibrand-Saint Oyant et al., 2018). The genomic physical positions are plotted on the *y*-axis, and the genetic position (cM) is plotted on the *x*-axis.

The linkage maps constructed with our two tetraploid garden rose mapping populations SWxBE *n* = 200 and BExMG *n* = 157 have total map lengths of 541.56 and 613.51 cM, average marker spacing of 0.11 and 0.10 cM, and the largest gap size of 8.42 and 3.37 cM, respectively (Table 2). Preferential pairing was detected, and recombination frequency estimates were corrected on LG 1 in the SWxBE population and LGs 1, 3, and 5 in the BExMG populations. The resulting maps from the two mapping populations, namely, SWxBE and BExMG, had good physical coverage and were collinear to the two *R. chinensis* genome assemblies (Hibrand-Saint Oyant et al., 2018; Raymond et al., 2018). The SWxBE and BExMG linkage maps display an inversion with respect to one of the *Rosa chinensis* genome assemblies (Hibrand-Saint Oyant et al., 2018), near *RoKSN* on LG 3 (Figure 2). This inversion was also observed in the comparison of the two *R. chinensis* genome assemblies (Smulders et al., 2019). The resulting phased maps were used in QTL mapping.

**TABLE 2:** Linkage map statistics of two tetraploid garden rose mapping populations genotyped using the WagRhSNP68k SNP array and mapped using the R-package polymapR.

Stormy weather x Brite eyes					Brite eyes x My girl			
LG	Markers	Map length	Average gap size	Largest gap size	Markers	Map length	Average gap size	Largest gap size
1	522	69.10	0.14	3.39	720	69.25	0.10	1.24
2	1289	84.11	0.07	1.46	1064	86.45	0.09	1.10
3	483	58.33	0.13	1.59	507	65.12	0.13	3.37
4	795	70.47	0.09	4.28	809	80.51	0.11	2.35
5	887	96.41	0.12	2.98	795	82.76	0.11	2.69
6	622	83.35	0.15	8.42	918	77.49	0.09	1.36
7	896	79.79	0.09	1.92	862	81.09	0.10	2.41
All	5494	541.56	0.11	8.42	5675	542.67	0.10	3.37

*Linkage maps were calculated using the Haldane mapping function which is the default in MDSmap implemented within R package polymapR. In addition, marker statistics generated using the R Shiny application The Genetic Map Comparator.*

### Variance Components

The genetic variance for RRD severity was 16.3% of the phenotypic variance measured over 3 years (Supplementary Table 1). The proportion of phenotypic variance due to environment (sets of observations) was 10.21 and 29.03% of the phenotypic variance was attributed to the compound symmetry between the plot and year. Variance component estimates were not calculated for RT-qPCR results as the design of the data collected did not allow for this calculation. Overall, this analysis showed that there was a genetic component to RRD resistance in our tetraploid populations giving us the confidence to pursue QTL analysis with our populations.

### Quantitative Trait Loci (QTL) Analysis

**FIGURE 3:**
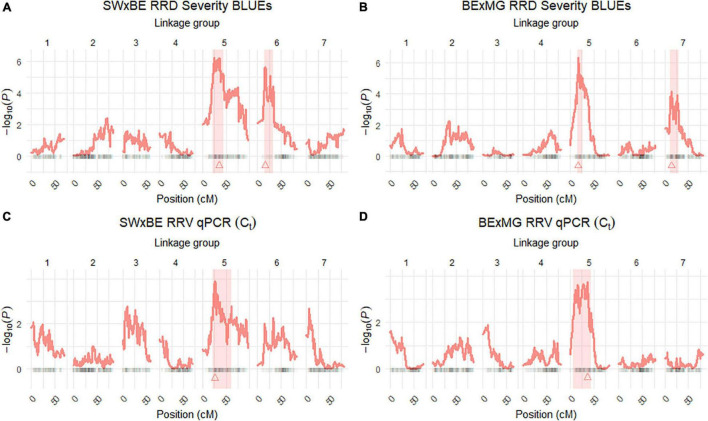
Quantitative trait locus (QTL) scans of **(A,B)** rose rosette disease (RRD) severity and **(C,D)** *Rose rosette emaravirus* (RRV) RT-qPCR detection using Ct values on two biparental tetraploid garden rose mapping populations. 95% confidence intervals are shaded while the QTL peak is represented by a triangle.

**FIGURE 4:**
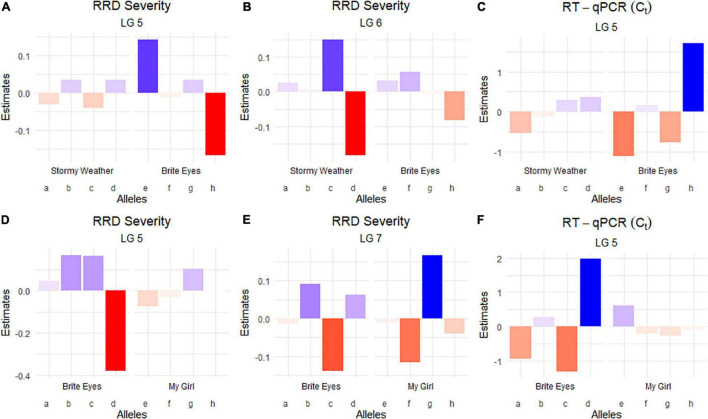
Quantitative trait locus allele effect estimates from QTLpoly on rose rosette disease severity and *Rose rosette emaravirus* RT-qPCR from two biparental tetraploid garden rose mapping populations. Bar plots indicate the direction of the QTL’s effect. **(A–C)** Allele effect plots of RRD severity for QTL on LGs 5 and 6, and for the RRV detection measured using RT-qPCR on LG 5 in the SWxBE population. **(D–F)** Allele effect plots of RRD severity for QTL on LGs 5 and 7, and for the RRV detection measured using RT-qPCR on LG 5 in the BExMG population.

The QTL analysis was performed using two methods. First, QTL analysis was conducted on two biparental populations separately using the R software package QTLpoly (da Silva Pereira et al., 2020), which utilizes a random effect multiple interval mapping approach for mapping multiple QTLs in autopolyploids. QTLs for RRD severity were detected on LGs 5 (qRRD.SWxBE-ch5, qRRD.BExMG-ch5), 6 (qRRD.SWxBE-ch6), and 7 (qRRD.BExMG-ch7) (Table 3; Figure 3; Supplementary Figures 2–4). Putative QTLs for RRD severity were detected on LG 2 in both families; however, the signal was too weak to further investigate. For each QTL, an estimated allele effect for each parental homolog was calculated at the QTL peak (Figure 4). For example, in Figure 4A, if a progeny carries an “h” parental homolog at the QTL location of 35.08 cM, this individual carries the resistance allele and thus should have lower RRD severity. The QTL on LG 5 was observed in both mapping populations and is inherited from the common parent Brite Eyes, which lowered the mean RRD severity score (Figure 4). The QTL on LG 6 was inherited from Stormy Weather and reduced the phenotypic RRD severity score while the QTL on LG 7 was inherited from My Girl, having the opposite effect of increased RRD severity (Figure 4).

**TABLE 3:** Quantitative trait loci (QTL) for rose rosette disease and *Rose rosette emaravirus* resistance in two tetraploid garden rose biparental mapping populations.

QTL*^a^*	Donor parent*^b^* (effect size)	LG	LOP*^c^*	Position (cM)*^d^*	Position (Mbp)*^e^*	PVE*^f^*	diaQTL position (Mbp)*^g^*
qRRD.SWxBE-ch5	BE↓ (−0.168)	5	6.15	35.08 (21.01–45.07)	6.04–25.08	0.20	9.54
qRRD.SWxBE-ch6	SW↓ (−0.183)	6	5.63	16.01 (14.27–33.64)	2.23–17.01	0.24	7.34
qRRV. SWxBE-ch5	BE↑ (+ 1.706)	5	3.89	26.15 (22.03–61.23)	6.41–44.20	0.18	9.18
qRRD.BExMG-ch5	BE↓ (−0.382)	5	6.31	17.10 (15.08–26.23)	7.62–19.69	0.40	9.54
qRRD.BExMG-ch7	MG↑ (+ 0.167)	7	4.15	15.03 (11.06–29.06)	2.63–13.59	0.14	NA
qRRV.BExMG-ch5	BE↑ (+ 1.968)	5	3.35	36.26 (6.19–44.14)	1.14–37.42	0.22	9.18

*^a^Name of QTL following naming conventions of the Genome Database for Rosaceae. RRD, rose rosette disease and RRV, Rose rosette emaravirus measured by quantitative polymerase chain reaction (RT-qPCR).*

*^b^Parent contributing allele affecting the phenotypic mean. Estimated from “qtl_effects” function in QTLpoly. Alleles affecting trait means are followed by the estimated direction of phenotypic effect of the presence of the parental allele.*

*^c^LOP calculated as -log(P-value) of the QTL peak. LOP for qRRV.BExMG-ch5 was calculated using relaxed P-values.*

*^d^QTL peak position followed by 1.5 LOD confidence intervals in parenthesis.*

*^e^Physical positions of markers within the 1.5 LOD confidence intervals. WagRhSNP 68k Axiom SNP array probes were aligned to the rose genome assembly by Hibrand-Saint Oyant et al., 2018.*

*^f^Percent variance explained (PVE) is estimated in QTLpoly as the QTL heritability ($h_{q}^{2}$) which is the ratio between the variance attributed to the QTL and the total variance.*

*^g^QTL peak position detected using diaQTL in million base pairs.*

The RRD severity BLUEs used for QTL mapping were the result of 4 sets of phenotypic observations while the *C*_*t*_ values used were an aggregate of observations such that when any genotype tested positive for the virus, it was no longer tested in subsequent RT-qPCR runs. In both mapping populations, the presence of a Brite Eyes allele increased the *C*_*t*_ value indicating lower amounts of virus. Interestingly, the allele effect estimate plots show that the same Brite Eyes homolog affects RRD severity and *C*_*t*_ values (Figure 4) indicating that both are controlled by the same or closely linked genetic factors. In addition to the *C*_*t*_ values, a binary presence/absence of virus was also used as a trait in the QTL analysis and gave similar results to the use of *C*_*t*_ values (Supplementary Figures 2, 3).

**FIGURE 5:**
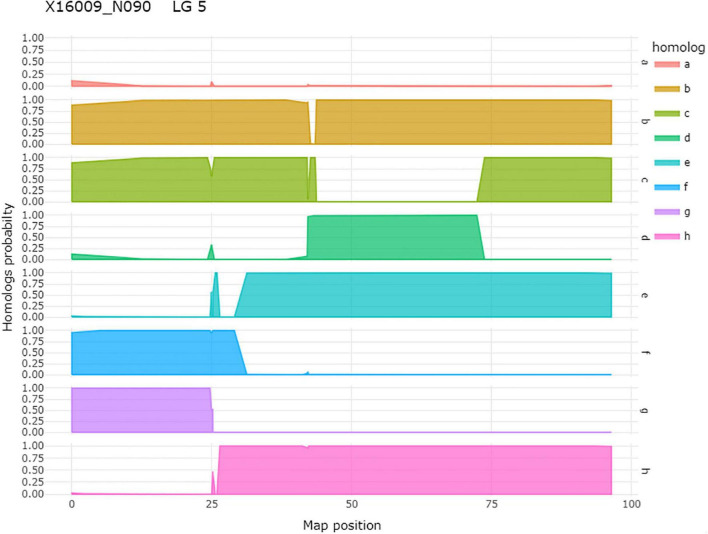
An example of homolog probabilities for each parental homolog for one individual, 16009-N090, of a tetraploid biparental mapping population on LG 5. On the *y*-axis is the probability that the individual carries the parental homolog. On the *x*-axis is the map position in centimorgan. In the case of this individual, the h-homolog at 35.08 cM carries the resistant allele for qRRD.SWxBE-ch5, and this is an individual that carries the resistant allele.

After QTL analysis, we identified individuals that carry resistance QTL using genotype and homolog probabilities calculated in the R software package MAPpoly. The allele effect plots from QTLpoly (Figure 4) identified which parental homologs contributed to resistance, which was used to identify all the progenies that carried the favorable parental homolog at the QTL peak. Homolog probabilities and recombination breakpoints were also visualized from the homolog probabilities (Figure 5). In the SWxBE population, we isolated 103 and 94 individuals carrying the resistant allele for the LGs 5 and 6 QTL, respectively, and 50 individuals with both resistance QTL. In the BExMG population, we identified 84 and 77 individuals that carry the QTL on LGs 5 and 7, respectively. There were 41 individuals that carried both QTLs (Table 4). The segregation ratios observed in these two populations reflect what is expected from a simplex by nulliplex segregation. We had 57 individuals that were genotyped but not phenotyped due to the number of clonal replicates available. Among those not phenotyped, we identified 30, 14, and 13 individuals that carried the RRD QTL resistance allele on LGs 5, 6, and 7, respectively. Resistant individuals will be useful in further breeding for RRD resistance. In addition to identifying resistant and susceptible individuals, we are able to use the linkage mapping data and the phased haplotype data to find markers, which track the resistant QTL in the progeny of future generations.

**TABLE 4:** Individuals from two biparental tetraploid garden rose mapping populations carrying quantitative trait loci (QTL) for rose rosette disease (RRD) severity.

QTL	Number of individuals carrying the QTL
**Results from QTL mapping from QTLpoly**	
LG 7	77
LG 7 (only)*	36
LG 5	187**
LG 5 (only)	96
LG 6	94
LG 6 (only)	44
LG 5 & LG 7	41
LG 5 & LG 6	50
**Results from diaQTL joint analysis**	
LG 5	190
LG 5 (only)	129
LG 6	112
LG 6 (only)	51
LG 5 & LG 6	61

*QTLs followed by (only) are the number of individuals that only carry that QTL and no other QTL, whereas the QTL without only contains individuals that may carry multiple QTLs due to independent segregation.

**103 in the Stormy Weather × Brite Eyes population and 84 in the Brite Eyes × My Girl population. RRD, rose rosette disease.

**FIGURE 6:**
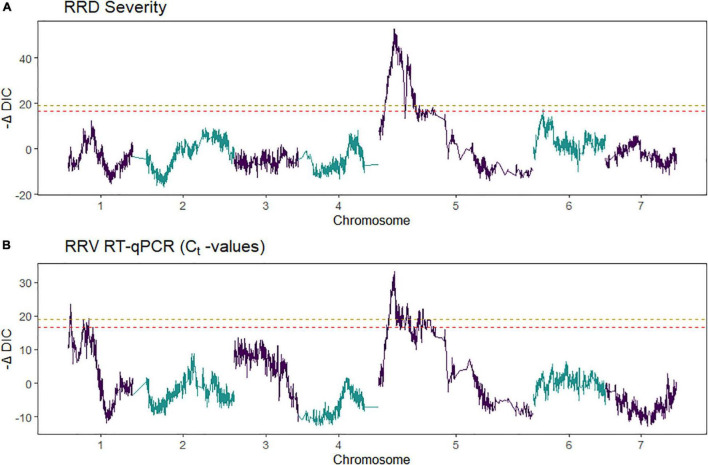
Quantitative trait locus scans from two tetraploid garden rose interconnected populations for **(A)** rose rosette disease (RRD) severity and **(B)** *C*_*t*_ values from R-software package diaQTL. On the *x*-axis is the genome position and on the *y*-axis is the relative strength of the QTL measured –ΔDIC, the test statistic used in detecting QTL in diaQTL. The peaks observed in the –ΔDIC profile are similar to other test statistics used to plot QTL profiles. The gold-colored line and red-colored line are alpha = 0.05 and 0.10 genome-wide significance, respectively.

**FIGURE 7:**
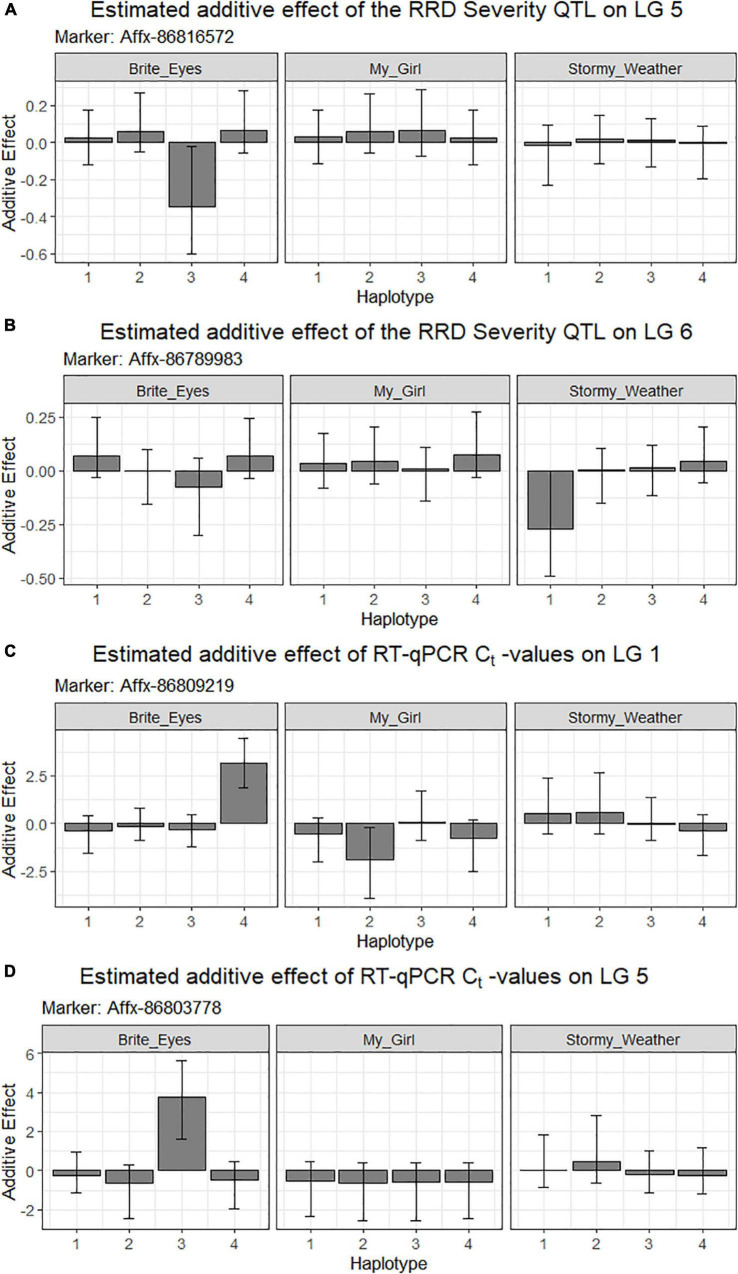
Estimated additive allele effects for **(A,B)** RRD severity and **(C,D)** RT-qPCR for RRV in two interconnected tetraploid garden rose mapping populations estimated from the R-software diaQTL. On the x-axis are the parental homologs, and the y-axis is the estimated additive effect of the parental homolog at the QTL position on the phenotype. For example: **(A)** the presence of the 3rd homolog in an individual has an estimated negative effect on the RRD severity observed.

Second, we jointly analyzed the interconnected families to reconstruct the parental haplotypes observed in the progeny using a Julia software package called PolyOrigin followed by an interconnected family QTL analysis using the R software package diaQTL. The use of this method allows for a better QTL resolution when looking at traits inherited from the common parent Brite Eyes as the homologs from that parent are represented more often than the Stormy Weather and My Girl homologs during the parental haplotype reconstruction. QTLs for RRD severity were detected at around 9.54 Mbp on LG 5 and around 7.34 Mbp on LG 6 (Figure 6). The QTL on LG 7 was not detected. For the *C*_*t*_ values, a QTL on LG 5 was detected in the same location and the same homolog as RRD severity (Figures 6, 7). Additionally, we found a QTL for *C*_*t*_ values on LG 1 from Brite Eyes. Using diaQTL, we identified 190 individuals with the LG 5 QTL and 112 individuals with the LG 6 QTL (Table 4). There were 61 individuals that carried both QTLs. Another interesting aspect of diaQTL is the ability to detect double reduction products. As both the QTLs on LGs 5 and 6 are near chromosomal ends and the probability of observing double reduction products increases as the distance from the centromere increases (Bourke et al., 2015), there is a good probability that there are individuals that may carry two copies of the parental haplotype due to double reduction. The analysis identified two individuals, 16009-N065 and 16401-N015, which have two resistance alleles from Brite Eyes on LG 5. Of these two individuals, 16401-N015 also had a QTL on LG 6. These two individuals would be useful LG 5 QTL donors for breeding as they already carry two copies of the favorable allele.

## Discussion

Recently, tools have been developed and are currently under active development for genetic analysis of autopolyploid organisms. Tools such as TetraploidMap and TetraploidSNPMap (Hackett and Luo, 2003; Hackett et al., 2017) have been available for some time but are not well suited for the large number of SNP markers obtained from the either sequencing technologies or SNP arrays. Recently, the availability of linkage mapping software such as polymapR (Bourke et al., 2017a) and MAPpoly (Mollinari and Garcia, 2019), along with their counterpart QTL mapping software polyqtlR (Bourke et al., 2021) and QTLpoly (da Silva Pereira et al., 2020), have allowed for efficient linkage mapping and QTL mapping of autotetraploid and autohexaploid crops with thousands of markers. New approaches utilize the interconnected nature of many breeding programs to bring better statistical power to haplotyping the common parental genotypes. One such program is polyOrigin (Zheng et al., 2021) and its counterpart interconnected QTL analysis software diaQTL (Amadeu et al., 2021). The software described in this study and used in this analysis has been previously used to create linkage maps and map QTL for various traits in other polyploid crops. The MAPpoly and QTLpoly software have been successfully used to map common scab resistance in autotetraploid potatoes (da Silva Pereira et al., 2021) and fruit firmness in autotetraploid blueberry (Cappai et al., 2020). This software has been used extensively to study autohexaploid sweet potatoes for root-knot nematode resistance (Oloka et al., 2021), carotenoid, and starch biosynthesis (Gemenet et al., 2020) and yield component traits (da Silva Pereira et al., 2020). Several autotetraploid rose maps have been constructed using polymapR (Bourke et al., 2017b; Zurn et al., 2018, 2020; Cheng et al., 2021; Yu et al., 2021) and QTL for stem prickles and flavonoid and carotenoid levels using polyqtlR (Bourke et al., 2021; Cheng et al., 2021). Interconnected population QTL analysis has been performed in autotetraploid potatoes using diaQTL analyzing tuber shape (Amadeu et al., 2021). Adding to the literature on utilizing newly developed software for autopolyploids is our detection of RRD resistance in autotetraploid garden roses. In this study, we used these various programs and approaches to study RRD resistance. The detection of the same QTL using multiple software shows the robustness of this new software that are being actively developed and gives us confidence in the detection of genetic factors affecting RRD resistance.

The linkage maps developed with our two mapping populations presented in this study are on par with the other published high-density SNP-based linkage maps (Bourke et al., 2017b; Zurn et al., 2018, 2020; Cheng et al., 2021; Yu et al., 2021), bringing the total number of tetraploid rose linkage maps reported to seven. The total lengths of our maps were similar to the K5 cut rose mapping population (568.67 cM) (Bourke et al., 2017b), as we also used the default Haldane mapping function and the polymapR implementation of MDSmap (Preedy and Hackett, 2016). Our maps were roughly 100 cM longer than the two garden rose mapping populations (421.92 and 405.42 cM) (Zurn et al., 2018, 2020) using the Kosambi mapping function. The linkage maps constructed with the Chinese tetraploid YS mapping population using whole-genome sequencing (Cheng et al., 2021) (1285.11 cM) and specific locus amplified fragment sequencing (SLAF-seq) (Yu et al., 2021) (1158.90 cM) were more than double the length of our maps. All the maps described above were constructed using R-package polymapR. All the linkage maps were similar in size and quality with the exception of the YS maps (Cheng et al., 2021; Yu et al., 2021). The difference in genotyping platforms may be one of the factors contributing to the discrepancies between the lengths of these maps as the maps that are similar in length were genotyped using the WagRhSNP 68K array while the YS mapping population was genotyped using whole-genome resequencing and SLAF-seq. We suspected that SNP array data have less genotyping error when compared with sequencing-derived data. Thus, the higher genotyping error rate may have contributed to the data containing artificial double recombinations resulting in inflated maps (Hackett and Broadfoot, 2003). Like the other five tetraploid rose mapping populations (Bourke et al., 2017b; Zurn et al., 2018, 2020; Cheng et al., 2021; Yu et al., 2021), which have been maintained and are used to study other traits, our two mapping populations present a good genetic resource to help dissect other traits of interest *via* future QTL studies.

Up to this point, there have been no genetic studies on resistance for RRV although work on resistance QTL for two emaraviruses, Pigeonpea sterility mosaic virus (Gnanesh et al., 2011; Saxena et al., 2017) and High Plains wheat mosaic virus (Marçcon et al., 1999), have been previously described. In this study, we have identified a partial resistance locus to the *Rose rosette emaravirus* in tetraploid roses on LG 5 inherited from Brite Eyes. Even though there was difficulty in phenotyping, as disease symptoms developed slowly over the 3-year trial, the QTL peaks were strong (3–7 LOD/LOP) and were consistently detected when using multiple software packages. In addition, the analysis from QTLpoly and diaQTL indicate that not only are the RRD severity and *C*_*t*_ value QTL co-localized but the same homolog from Brite Eyes affects these two traits. Since the QTL on LG 5 was detected in both mapping populations, with the QTL coming from the common parent Brite Eyes, we are confident that this is an important locus for RRD resistance which we would like to further dissect in future studies. Furthermore, we were able to identify markers that can be used to track the QTL in further crosses with this germplasm.

In a preliminary search for candidate genes for RRD resistance, we searched between 7.64 and 13.08 Mbp in the *Rosa chinensis* Genome version 1.0 (Hibrand-Saint Oyant et al., 2018) for any annotated genes that may be of interest to viral resistance. We found roughly 40 genes, which were associated with ribosomal functions, ∼20 genes with polymerase-associated genes, 7 nuclear binding site – leucine-rich repeat (NBS-LRR) related genes, 6 WRKY transcription factor related genes, and a couple of eukaryotic translation initiation factors (eIF). Translation and replication factors along with NBS-LRR, WRKY, and eIF genes are some of the broad families of genes that affect general disease and viral resistance in various organisms (DeYoung and Innes, 2006; Wang et al., 2013; Chen et al., 2019). Larger mapping populations are needed to fine map the region of interest so we can hone in on candidate genes. Another consideration is that our tetraploid garden rose populations may have a genetically different background compared with the doubled haploid used for the *R. chinensis* reference assembly which provides another obstacle to narrowing in on candidate genes.

The collinearity of the RRD severity scores and RT-qPCR QTL along with the allele effect plots showing that the same homolog is responsible for both traits provides supporting evidence that the same genetic factor affecting the RRD severity phenotype is the same factor affecting viral titer in the plant. Markers associated with the QTL for RRD on either side of the QTL (haplotypes) will be useful in marker-assisted breeding as assays can be designed for tracking the inheritance of the QTL. In the case of RRD where phenotyping for resistance can take 3–4 years, a genetic test for resistant progeny would be a very useful tool to accelerate the development of roses resistant to RRD.

**FIGURE 8:**
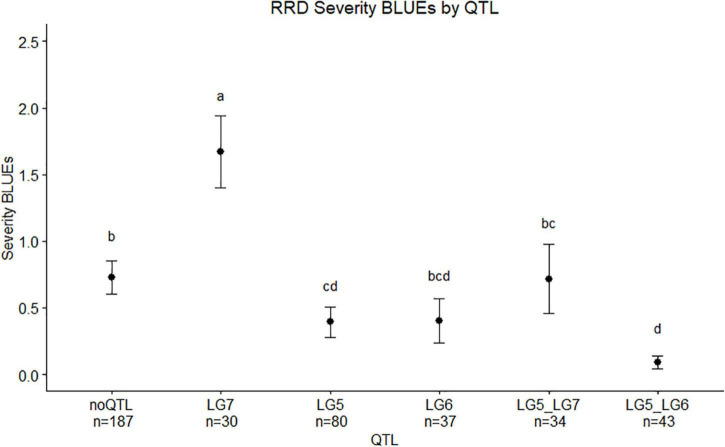
Means separation and 0.95 confidence intervals of RRD severity best linear unbiased estimates (BLUEs) measured in two tetraploid biparental garden rose mapping populations grouped by QTL carried by progeny. Groups with the same letters are not significantly different from each other using Tukey’s test (α = 0.05). Individuals shown in this study are from r-packages MAPpoly and QTLpoly (note: the sample numbers depicted in this study represent only the individuals that were phenotyped).

We observed twice the amount of RRD severity in the BExMG family compared with the SWxBE family (Figure 1; Table 1). While both families have the QTL for reduced susceptibility inherited from Brite Eyes on LG 5, other QTLs also associated with the trait can have effects on the overall susceptibility of the genotypes. The two QTLs in the SWxBE family on LGs 5 and 6 both reduce the amount of RRD severity while the two QTLs in the BExMG family on LGs 5 and 7 have opposing effects. The opposing severity effect from LG 7 to the effect of LG 5 along with the SWxBE population segregating for two QTLs for reduced susceptibility is most likely the reason for the difference in susceptibility between the families. Figure 8 shows the mean RRD severity BLUEs of individuals with the LG 7 QTL having the highest mean RRD severity scores. It is possible that under greater infection pressure, a greater difference between the two families will become apparent.

There are advantages of using different software for the detection of QTL as they complement each other to create a more complete picture of the genetic landscape of the germplasm used. MAPpoly and QTLpoly helped isolate the parental homologs which carry the disease resistance and diaQTL allowed harnessing the interconnectedness of the populations. This software provides, for example, a confidence interval for where the QTL lies in the homolog where a QTL is detected. Since all genotypes are haplotyped before a QTL scan is performed, this analysis also yields information on the source (homolog and parent) of the QTL and the associated SNP marker haplotype (markers in the confidence interval). Using diaQTL, we identified two individuals which had two copies of the favorable allele due to double reduction.

As discussed above, an important output of this study is the identification of individuals that may be useful in breeding since they carry the various SNP-tagged QTL combinations. Assuming sib or self-pollination in this germplasm is possible and inbreeding depression will not be a major obstacle, intercrossing or self-pollinating individuals with the LG 5 QTL in a duplex state should result in the production of a triplex with an 8:36 chance and a quadruplex (homozygous for the QTL) with a 1:36 chance. It will be interesting to see the level of resistance enhancement that can be achieved by increasing the copy number of the LG 5 QTL. Also, individuals with the LG 5 QTL in the duplex state are good QTL donors for breeding. Assuming normal transmission of LG5 QTL, it is expected that ∼85% of progenies in crosses involving these QTL carriers will inherit at least one copy of this QTL. This should allow a relatively rapid transfer of this QTL to other germplasm.

## Conclusion

The two tetraploid mapping populations presented in this study were used to estimate variance components of RRD phenotypic variation, create new linkage maps, map the first RRD/RRV QTL in tetraploid roses, and identify resistant individuals, which will be useful in future breeding. Future work is needed to validate the QTL reported in this study. Furthermore, additional studies are needed to narrow down the LG 5 QTL region inherited from Brite Eyes in order to identify candidate genes. This study helps the rose industry fight against the devastating RRV by helping us better understand the genetics behind RRV resistance which ultimately aids in the creation of new RRV resistant commercial cultivars.

## Data Availability Statement

The raw data supporting the conclusions of this article will be made available by the authors, without undue reservation.

## Author Contributions

DB conceived and designed the project. JL made the crosses, propagated the mapping populations, collected the tissue, performed genetic mapping, and QTL analysis. EY helped with the crosses, propagation, and planting of the experiment. SC and MW performed field-based phenotypic assessments, RT-qPCR assays, and maintenance of the experimental fields. PK provided funding and technical expertise for rose DNA extractions. OR-L provided guidance in the interpretation of QTL analysis results. All authors contributed to the article and approved the submitted version.

## Conflict of Interest

The authors declare that the research was conducted in the absence of any commercial or financial relationships that could be construed as a potential conflict of interest.

## Publisher’s Note

All claims expressed in this article are solely those of the authors and do not necessarily represent those of their affiliated organizations, or those of the publisher, the editors and the reviewers. Any product that may be evaluated in this article, or claim that may be made by its manufacturer, is not guaranteed or endorsed by the publisher.

## Supplementary Material

Supplementary Figure 1 Heatmaps of rose rosette disease (RRD) severity measured in two tetraploid garden rose populations throughout the field over the 4 sets of phenotypic observations. The *x*-axis contains rows, and the *y*-axis is the plant number within the row. The gray background of plots signifies missing plants or inoculum rows (row 1 and row 9). Severity gradients white to red are severity scores of both populations.

Supplementary Figure 2 Quantitative trait locus (QTL) profiles of RRD severity in a tetraploid garden rose mapping population Brite Eyes × My Girl on a yearly basis, across years as best linear unbiased estimates (BLUEs), qPCR results using *C*_*t*_ value, and as a binary trait (presence or absence of virus detection).

Supplementary Figure 3 Quantitative trait locus profiles of RRD severity in a tetraploid garden rose mapping population Stormy Weather × Brite Eyes on a yearly basis, across years as BLUEs, qPCR results using *C*_*t*_ value, and as a binary trait (presence or absence of virus detection).

Supplementary Figure 4 Rose rosette disease QTL detected in two interconnected tetraploid rose mapping populations using QTLpoly. Positions on the map are in Mbp, and the QTLs are color-coded for which population they were detected. The length of the QTL bars represents the 95% confidence interval for the QTL when looking at the physical location of markers in the confidence interval. QTL with the labels qRRD refers to QTL for rose rosette disease severity scores, and qRRV refers to QTL detected using the *C*_*t*_ values from the RT-qPCR detection of *Rose rosette emaravirus*.

Supplementary Table 1 Variance component estimates for rose rosette disease severity scores in two tetraploid rose biparental mapping populations phenotyped in Crossville, TN, United States.

Supplementary File 1 Brite Eyes × My Girl linkage map.

Supplementary File 2 Stormy Weather × Brite Eyes linkage map.

## References

[B1] Allington W. B. Staples R. Viehmeyer G. (1968). Transmission of Rose Rosette Virus by the Eriophyid Mite *Phyllocoptes fructiphilus*. *J. Econ. Entomol.* 61 1137–1140. 10.1093/jee/61.5.1137

[B2] Amadeu R. R. Muñoz P. R. Zheng C. Endelman J. B. (2021). QTL mapping in outbred tetraploid (and diploid) diallel populations. *Genetics* 219:iyab124. 10.1093/genetics/iyab124 34740237 PMC8570786

[B3] Amrine J. W. Jr. Hindal D. F. Stasny T. A. Williams R. L. Coffman C. C. (1988). Transmission of the rose rosette disease agent to *Rosa multiflora* by *Phyllocoptes fructiphilus* (Acari: Eriophyidae). *Entomol. News U.S.A*. 99 239-252.

[B4] Amrine J. W. (1996). “4.1.2 *Phyllocoptes fructiphilus* and biological control of multiflora rose,” in *World Crop Pests* Eriophyoid Mites Their Biology, Natural Enemies and Control, eds Lindquist E. E. Sabelis M. W. Bruin J. (Amsterdam: Elsevier), 741–749. 10.1016/S1572-4379(96)80050-9

[B5] Bourke P. Geest G. van, Voorrips R. E. Jansen J. Kranenburg T. (2017a). polymapR: linkage analysis and genetic map construction from F1 populations of outcrossing polyploids. *bioRxiv*. [preprint]. 10.1101/228817 PMC6184683 29722786

[B6] Bourke P. M. Arens P. Voorrips R. E. Esselink G. D. Koning-Boucoiran C. F. S. van’t Westende W. P. C. (2017b). Partial preferential chromosome pairing is genotype dependent in tetraploid rose. *Plant J.* 90 330–343. 10.1111/tpj.13496 28142191

[B7] Bourke P. M. Voorrips R. E. Hackett C. A. van Geest G. Willemsen J. H. Arens P. (2021). Detecting quantitative trait loci and exploring chromosomal pairing in autopolyploids using polyqtlR. *Bioinformatics* 37 3822–3829. 10.1093/bioinformatics/btab574 34358315 PMC8570814

[B8] Bourke P. M. Voorrips R. E. Visser R. G. F. Maliepaard C. (2015). The double-reduction landscape in tetraploid potato as revealed by a high-density linkage map. *Genetics* 201 853–863. 10.1534/genetics.115.181008 26377683 PMC4649655

[B9] Butler D. G. Cullis B. R. Gilmour A. R. Gogel B. J. Thompson R. (2017). *ASReml-R Reference Manual Version 4.* Hemel Hempstead: VSN International Ltd.

[B10] Byrne D. H. Klein P. Yan M. Young E. Lau J. Ong K. (2018). Challenges of Breeding Rose Rosette–resistant Roses. *HortScience* 53 604–608. 10.21273/HORTSCI12553-17 35581909

[B11] Cappai F. Amadeu R. R. Benevenuto J. Cullen R. Garcia A. Grossman A. (2020). High-Resolution Linkage Map and QTL Analyses of Fruit Firmness in Autotetraploid Blueberry. *Front. Plant Sci.* 11:562171. 10.3389/fpls.2020.562171 33304360 PMC7701094

[B12] Chen X. Li C. Wang H. Guo Z. (2019). WRKY transcription factors: evolution, binding, and action. *Phytopathol. Res.* 1:13. 10.1186/s42483-019-0022-x

[B13] Cheng B. Wan H. Han Y. Yu C. Luo L. Pan H. (2021). Identification and QTL analysis of flavonoids and carotenoids in tetraploid roses based on an ultra-high-density genetic map. *Front. Plant Sci.* 12:682305. 10.3389/fpls.2021.682305 34177997 PMC8226220

[B14] Conners I. L. (1941). Twentieth annual report of the Canadian plant disease survey, 1940. *Twent. Annu. Rep. Can. Plant Dis. Surv* 1940:98.

[B15] da Silva Pereira G. Gemenet D. C. Mollinari M. Olukolu B. A. Wood J. C. (2020). Multiple QTL mapping in autopolyploids: A random-effect model approach with application in a hexaploid sweetpotato full-sib population. *Genetics* 215 579–595. 10.1534/genetics.120.303080 32371382 PMC7337090

[B16] da Silva Pereira G. Mollinari M. Qu X. Thill C. Zeng Z.-B. (2021). Quantitative Trait Locus Mapping for Common Scab Resistance in a Tetraploid Potato Full-Sib Population. *Plant Dis.* 105 3048–3054. 10.1094/PDIS-10-20-2270-RE 33728960

[B17] DeYoung B. J. Innes R. W. (2006). Plant NBS-LRR proteins in pathogen sensing and host defense. *Nat. Immunol.* 7 1243–1249. 10.1038/ni1410 17110940 PMC1973153

[B18] Dobhal S. Olson J. D. Arif M. Garcia Suarez J. A. Ochoa-Corona F. M. (2016). A simplified strategy for sensitive detection of Rose rosette virus compatible with three RT-PCR chemistries. *J. Virol. Methods* 232 47–56. 10.1016/j.jviromet.2016.01.013 26850142

[B19] Doudrick R. L. White J. A. Millikan D. F. (1987). Graft and mechanical transmission of the rose rosette agent. *Trans. Mo. Acad. Sci. U.S.A*. 21, 81–86.

[B20] Gemenet D. C. da Silva, Pereira G. De Boeck B. Wood J. C. Mollinari M. (2020). Quantitative trait loci and differential gene expression analyses reveal the genetic basis for negatively associated β-carotene and starch content in hexaploid sweetpotato [Ipomoea batatas (L.) Lam.]. *Theor. Appl. Genet.* 133 23–36. 10.1007/s00122-019-03437-7 31595335 PMC6952332

[B21] Gnanesh B. N. Bohra A. Sharma M. Byregowda M. Pande S. Wesley V. (2011). Genetic mapping and quantitative trait locus analysis of resistance to sterility mosaic disease in pigeonpea [Cajanus cajan (L.) Millsp.]. *Field Crops Res.* 123 53–61. 10.1016/j.fcr.2011.04.011

[B22] Gudin S. (2000). Rose: genetics and breeding. *Plant Breed. Rev.* 17 159–190.

[B23] Hackett C. A. Boskamp B. Vogogias A. Preedy K. F. Milne I. (2017). TetraploidSNPMap: Software for Linkage Analysis and QTL Mapping in Autotetraploid Populations Using SNP Dosage Data. *J. Hered.* 108 438–442. 10.1093/jhered/esx022

[B24] Hackett C. A. Broadfoot L. B. (2003). Effects of genotyping errors, missing values and segregation distortion in molecular marker data on the construction of linkage maps. *Heredity* 90 33–38. 10.1038/sj.hdy.6800173 12522423

[B25] Hackett C. A. Luo Z. W. (2003). TetraploidMap: Construction of a Linkage Map in Autotetraploid Species. *J. Hered.* 94 358–359. 10.1093/jhered/esg066 12920109

[B26] Hibrand-Saint Oyant L. Ruttink T. Hamama L. Kirov I. Lakhwani D. Zhou N.-N. (2018). A high-quality sequence of *Rosa chinensis* to elucidate genome structure and ornamental traits. *Nat. Plants* 4 473–484. 10.1101/254102 29892093 PMC6786968

[B27] Holtz Y. David J. L. Ranwez V. (2017). The genetic map comparator: a user-friendly application to display and compare genetic maps. *Bioinforma. Oxf. Engl.* 33 1387–1388. 10.1093/bioinformatics/btw816 28453680

[B28] Jian H. Zhang H. Tang K. Li S. Wang Q. Zhang T. (2010). Decaploidy in Rosa praelucens Byhouwer (Rosaceae) Endemic to Zhongdian Plateau. *Yunnan China. Caryologia* 63 162–167. 10.1080/00087114.2010.10589722

[B29] Koning-Boucoiran C. F. S. Esselink G. D. Vukosavljev M. Van ’t Westende W. P. C. Gitonga V. W. Krens F. A. (2015). Using RNA-Seq to assemble a rose transcriptome with more than 13,000 full-length expressed genes and to develop the WagRhSNP 68k Axiom SNP array for rose (Rosa L.). *Front. Plant Sci.* 6:249. 10.3389/fpls.2015.00249 25954285 PMC4404716

[B30] Laney A. G. Keller K. E. Martin R. R. Tzanetakis I. E. Y. (2011). A discovery 70 years in the making: characterization of the Rose rosette virus. *J. Gen. Virol.* 92, 1727–1732. 10.1099/vir.0.031146-0 21471323

[B31] Marçcon A. Kaeppler S. M. Jensen S. G. Senior L. Stuber C. (1999). Loci controlling resistance to High Plains Virus and Wheat Streak Mosaic Virus in a B73 × Mo17 population of maize. *Crop Sci.* 39 1171–1177. 10.2135/cropsci1999.0011183X003900040037x

[B32] Mielke-Ehret N. Mühlbach H.-P. (2012). *Emaravirus*: A novel genus of multipartite, negative strand RNA plant viruses. *Viruses* 4 1515–1536. 10.3390/v4091515 23170170 PMC3499817

[B33] Mollinari M. Garcia A. A. F. (2019). Linkage analysis and haplotype phasing in experimental autopolyploid populations with high ploidy level using Hidden Markov Models. *G3* 9 3297–3314. 10.1534/g3.119.400378 31405891 PMC6778803

[B34] Oloka B. M. da Silva, Pereira G. Amankwaah V. A. Mollinari M. Pecota K. V. (2021). Discovery of a major QTL for root-knot nematode (Meloidogyne incognita) resistance in cultivated sweetpotato (Ipomoea batatas). *Theor. Appl. Genet.* 134 1945–1955. 10.1007/s00122-021-03797-z 33813604 PMC8263542

[B35] Pemberton H. B. Ong K. Windham M. Olson J. Byrne D. H. (2018). What is Rose Rosette Disease? *HortScience* 53 592–595. 10.21273/HORTSCI12550-17 35581909

[B36] Preedy K. F. Hackett C. A. (2016). A rapid marker ordering approach for high-density genetic linkage maps in experimental autotetraploid populations using multidimensional scaling. *Theor. Appl. Genet.* 129 2117–2132. 10.1007/s00122-016-2761-8 27502200

[B37] Raymond O. Gouzy J. Just J. Badouin H. Verdenaud M. Lemainque A. (2018). The Rosa genome provides new insights into the domestication of modern roses. *Nat. Genet.* 50 772–777. 10.1038/s41588-018-0110-3 29713014 PMC5984618

[B38] Saxena R. K. Kale S. M. Kumar V. Parupali S. Joshi S. Singh V. (2017). Genotyping-by-sequencing of three mapping populations for identification of candidate genomic regions for resistance to sterility mosaic disease in pigeonpea. *Sci. Rep.* 7:1813. 10.1038/s41598-017-01535-4 28500330 PMC5431754

[B39] Smulders M. J. M. Arens P. Bourke P. M. Debener T. Linde M. Riek J. D. (2019). In the name of the rose: a roadmap for rose research in the genome era. *Hortic. Res.* 6:65. 10.1038/s41438-019-0156-0 31069087 PMC6499834

[B40] USDA NASS (2019). *2019 Census of horticultural specialties.* Available online at: https://www.nass.usda.gov/Publications/AgCensus/2017/Online_Resources/Census_of_Horticulture_Specialties/index.php (Accessed on Jan 20 2022)

[B41] Voorrips R. E. Gort G. Vosman B. (2011). Genotype calling in tetraploid species from bi-allelic marker data using mixture models. *BMC Bioinform.* 12:172. 10.1186/1471-2105-12-172 21595880 PMC3121645

[B42] Wang X. Kohalmi S. E. Svircev A. Wang A. Sanfaçon H. Tian L. (2013). Silencing of the host factor eIF(iso)4E gene confers plum pox virus resistance in plum. *PLoS One* 8:e50627. 10.1371/journal.pone.0050627 23382802 PMC3557289

[B43] Windham A. Windham M. Hale F. (2019). *Early Detection of Rose Rosette Disease.* Available online at: https://extension.tennessee.edu/publications/Documents/SP806.pdf (accessed February 14, 2022).

[B44] Windham M. Windham A. Hale F. Amrine J. Jr. (2014). Observations on rose rosette disease. *Am. Rose* 42 56–62.

[B45] Yan M. Byrne D. H. Klein P. E. Yang J. Dong Q. Anderson N. (2018). Genotyping-by-sequencing application on diploid rose and a resulting high-density SNP-based consensus map. *Hortic. Res.* 5 1–14. 10.1038/s41438-018-0021-6 29619228 PMC5878828

[B46] Yu C. Wan H. Bourke P. M. Cheng B. Luo L. Pan H. (2021). High density genetic map and quantitative trait loci (QTLs) associated with petal number and flower diameter identified in tetraploid rose. *J. Integr. Agric.* 20 1287–1301. 10.1016/S2095-3119(20)63416-5

[B47] Zheng C. Amadeu R. R. Munoz P. R. Endelman J. B. (2021). Haplotype reconstruction in connected tetraploid F1 populations. *Genetics* 219:iyab106. 10.1093/genetics/iyab106 34849879 PMC8633103

[B48] Zlesak D. C. (2007). *“Rose,” in Flower Breeding and Genetics.* Dordrecht: Springer, 695–740. 10.1007/978-1-4020-4428-1_26

[B49] Zurn J. D. Zlesak D. C. Holen M. Bradeen J. M. Hokanson S. C. Bassil N. V. (2018). Mapping a novel black spot resistance locus in the climbing rose Brite Eyes™ (‘RADbrite’). *Front. Plant Sci.* 9:1730. 10.3389/fpls.2018.01730 30534133 PMC6275305

[B50] Zurn J. D. Zlesak D. C. Holen M. Bradeen J. M. Hokanson S. C. Bassil N. V. (2020). Mapping the black spot resistance locus Rdr3 in the shrub rose ‘George Vancouver’ allows for the development of improved diagnostic markers for DNA-informed breeding. *Theor. Appl. Genet* 133 2011–2020. 10.1007/s00122-020-03574-4 32166372

