# Supplementary figures and images for: Rose Rosette Disease Resistance Loci Detected in Two Interconnected Tetraploid Garden Rose Populations
Source: Front Plant Sci. 2022 Jul 7;13:916231. doi: 10.3389/fpls.2022.916231 (PMC9302375; doi:10.3389/fpls.2022.916231)

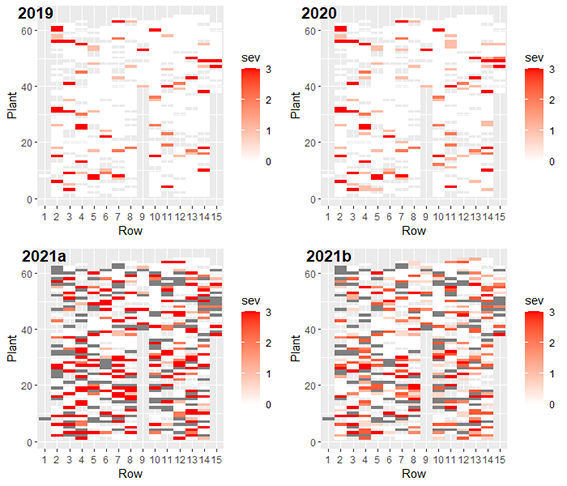

Supplement: Supplementary Figure 1 — Heatmaps of rose rosette disease (RRD) severity measured in two tetraploid garden rose populations throughout the field over the 4 sets of phenotypic observations. The x-axis contains rows, and the y-axis is the plant number within the row. The gray background of plots signifies missing plants or inoculum rows (row 1 and row 9). Severity gradients white to red are severity scores of both populations. [file Image_1.TIFF]

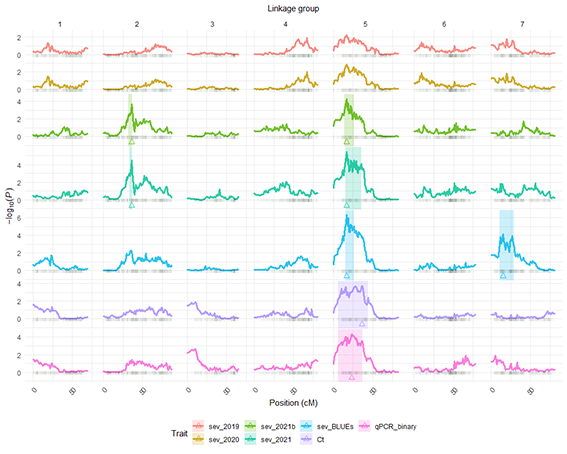

Supplement: Supplementary Figure 2 — Quantitative trait locus (QTL) profiles of RRD severity in a tetraploid garden rose mapping population Brite Eyes × My Girl on a yearly basis, across years as best linear unbiased estimates (BLUEs), qPCR results using Ct value, and as a binary trait (presence or absence of virus detection). [file Image_2.TIFF]

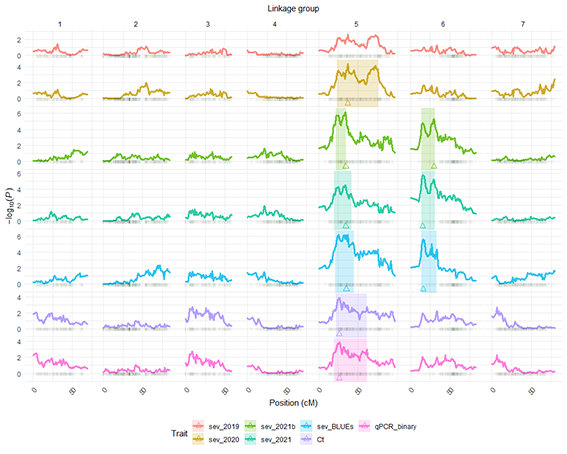

Supplement: Supplementary Figure 3 — Quantitative trait locus profiles of RRD severity in a tetraploid garden rose mapping population Stormy Weather × Brite Eyes on a yearly basis, across years as BLUEs, qPCR results using Ct value, and as a binary trait (presence or absence of virus detection). [file Image_3.TIFF]

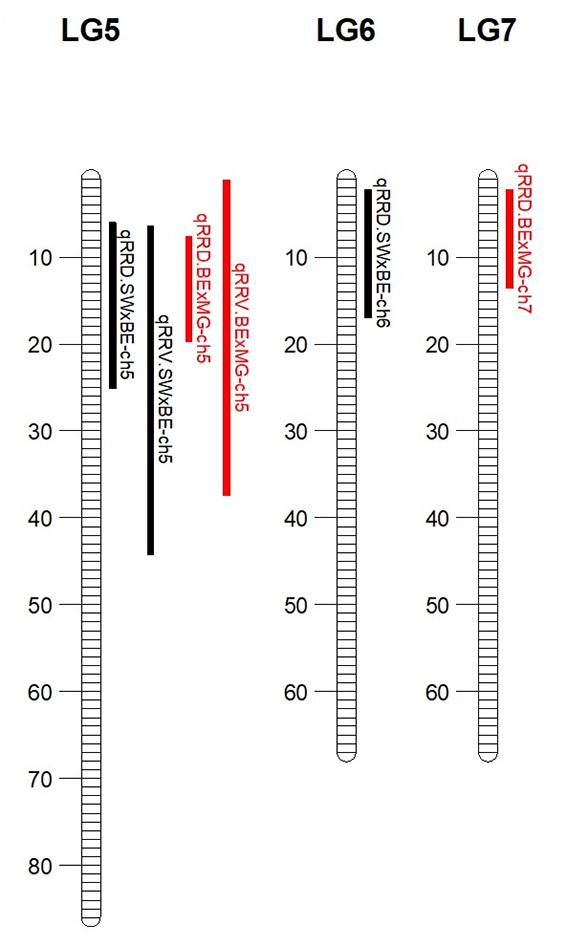

Supplement: Supplementary Figure 4 — Rose rosette disease QTL detected in two interconnected tetraploid rose mapping populations using QTLpoly. Positions on the map are in Mbp, and the QTLs are color-coded for which population they were detected. The length of the QTL bars represents the 95% confidence interval for the QTL when looking at the physical location of markers in the confidence interval. QTL with the labels qRRD refers to QTL for rose rosette disease severity scores, and qRRV refers to QTL detected using the Ct values from the RT-qPCR detection of Rose rosette emaravirus. [file Image_4.TIFF]
