# Supplementary material for: Rose Rosette Disease Resistance Loci Detected in Two Interconnected Tetraploid Garden Rose Populations
Source: Front Plant Sci. 2022 Jul 7;13:916231. doi: 10.3389/fpls.2022.916231 (PMC9302375; doi:10.3389/fpls.2022.916231)
Supplement: Supplementary Table 1 — Variance component estimates for rose rosette disease severity scores in two tetraploid rose biparental mapping populations phenotyped in Crossville, TN, United States. [file Table_1.docx]

**Supplementary Table 1**. Variance components estimates for rose rosette disease severity scores in two tetraploid rose bi-parental mapping populations phenotyped in Crossville, TN.

|  | **Variance components**^a^ | **Percent of variance**^b^ |
| --- | --- | --- |
| **Genotype** | 0.271 (0.045)*** | 16.29 |
| **Observation Set**^c^ | 0.170 (0.139)*** | 10.21 |
| **Rep(Year)** | 0.000 (0.001) | 0.00 |
| **Genotype x Year** | 0.140 (0.019)*** | 8.41 |
| **Residual** | 0.600 (0.037) | 36.06 |
| **Compound symmetry between plot and year** | 0.483 (0.036) | 29.03 |
| **Total** | 1.663 |  |

^a^Variance components and standard error in parenthesis.

^b^ Variance components estimated by taking each variance divided by total variance.

^c^Observation set defined as each set of phenotypic observations, namely years 2019, 2020, and two sets of observations in 2021.

^*^, ^**^, ^***^ Variance components are significant at P≤0.05, 0.01, or 0.001, respectively using using log-likelihood ratio tests.
